# Evaluating the Cardiovascular Benefits of Innovator Semaglutide 2.4 mg (Recombinant DNA (r-DNA)) in Patients With Overweight or Obesity: A Critical Review of Current Evidence

**DOI:** 10.7759/cureus.111322

**Published:** 2026-06-22

**Authors:** Brij Teli, Sunil Sathe, Ravi Kalra, Vivek Mahajan, Ajit Mehta, Rituprana Shinde, Brijesh Kunwar, Pravin R Kahale, Amit Sinkar, Priya Palimkar, Mohan Magdum, Vaishali Deshmukh, Varun Nivargi, Jignesh Parikh, Suraj Patil, Ranjeet Patil, Akshay Kashid, Amit Thopte, Rahul Sawant, Sachin Lakade, Jagjeet Deshmukh, Krishna Dhoot, Bhushan Bari, Anesh Jain, Naresh Munot, Vishnu Mane, Rahul Patil, Kaushik Sheth, Omkar Thopte, Vikrant Khese, Vivek Arya, Shrikant Somani, DB Gaware, Vivek Patel, Vahid Bharmal, Abhijit Khadtare, Anand Ahuja, Satish Sawant, Abhishek Bhargava, Prashant Rajput

**Affiliations:** 1 Endocrinology, Diabetes and Metabolism, Granthi-Diabetes, Endocrine and Obesity Center (Unit of 'Aumkar Clinic'), Rajkot, IND; 2 Cardiology, Cardiac Care and Counselling Center, Pune, IND; 3 Interventional Cardiology, Bharati Vidyapeeth (Deemed to be University) Medical College & Hospital, Pune, IND; 4 Interventional Cardiology, Fortis Hospital, Mumbai, IND; 5 Interventional Cardiology, Jehangir Hospital/Heart Smart Clinic, Pune, IND; 6 Interventional Cardiology, Pune Heart Failure Clinic, Pune, IND; 7 Interventional Cardiology, Medicover Hospital, Mumbai, IND; 8 Interventional Cardiology, Kokilaben Hospital, Mumbai, IND; 9 Interventional Cardiology, Sinkar Cardiac Care, Pune, IND; 10 Cardiology, Sahyadri Super Speciality Hospital, Pune, IND; 11 Cardiology, MJM Hospital, Pune, IND; 12 Diabetes and Endocrinology, Deenanath Mangeshkar Hospital and Research Center, Pune, IND; 13 Interventional Cardiology, Joshi Hospital, Pune, IND; 14 Cardiology, Krishna Hospital, Pune, IND; 15 Cardiology, Medicover Hospital, Pune, IND; 16 Cardiology, Baner Heart Institute, Pune, IND; 17 Cardiology, Manipal Hospital, Pune, IND; 18 Cardiology, Dr. Thopte’s Heart Care Clinic, Pune, IND; 19 Cardiology, Hridaymitra Cardia Clinic, Pune, IND; 20 Cardiology, Hrudaysparsh Heart Care Centre, Pune, IND; 21 Cardiology, Dr. Bari's Heart and Sonography Clinic, Pune, IND; 22 Cardiology, Beats and Bites Superspeciality Clinic, Pune, IND; 23 Cardiology, Munot Heart Care and Research Centre, Pune, IND; 24 Cardiology, Lotus Heart Care Centre, Pune, IND; 25 Cardiology, Hridayam Heartcare, Pune, IND; 26 Cardiology, Ruby Hall Clinic, Pune, IND; 27 Cardiology, Heart Beat Foundation, Pune, IND; 28 Cardiology, Vighnaharta Heart Care Centre, Pune, IND; 29 Endocrinology and Diabetes, Center for Endocrine Disease and Diabetes, Ahmedabad, IND; 30 Endocrinology and Diabetes, Somani Diabetes and Endocrine Care Center, Ahmedabad, IND; 31 Cardiology, Cardi Care Clinic, Pune, IND; 32 Endocrinology, Marengo Care Institute of Medical Sciences (CIMS) Hospital, Ahmedabad, IND; 33 Endocrinology and Diabetes, Bhailal Amin General Hospital, Vadodara, IND; 34 Cardiovascular Disease, Rhythm Heart Institute, Vadodara, IND; 35 Interventional Cardiology, Sawant Heart Clinic, Pune, IND; 36 Cardiology, Precision Medical Clinic, Mumbai, IND; 37 Nephrology, Gleneagles Hospital, Mumbai, IND

**Keywords:** cardiovascular risk reduction, hfpef, obesity without diabetes, select trial, semaglutide 2.4 mg

## Abstract

Obesity and cardiovascular (CV) disease (CVD) are tightly intertwined global epidemics, with excess adiposity now recognized as a major, modifiable driver of atherosclerotic events, heart failure, and mortality. Despite advances in cardiometabolic care, residual CV risk remains high in people with obesity, underscoring the need for therapies that both reduce body weight and directly modify CV risk. Semaglutide, a long-acting glucagon-like peptide-1 receptor agonist (GLP-1RA), has emerged as a pivotal agent across the cardiometabolic spectrum, culminating in the SELECT trial, which demonstrated a reduction in three-point major adverse CV events (3P-MACE) in people with obesity and established CVD but without diabetes. Complementary evidence from heart failure with preserved ejection fraction (HFpEF) trials and real-world studies further supports a broad CV and functional benefit profile. This narrative review synthesizes data from randomized controlled trials (RCTs) and real-world evidence (RWE) on Wegovy® (semaglutide 2.4 mg, a recombinant DNA-derived GLP-1RA) in obesity with CVD, with a focus on SELECT, mediation analyses suggesting weight-independent CV effects, and outcomes in obesity-related HFpEF. We also discuss guideline positioning, regulatory milestones-including the 2025 Central Drugs Standard Control Organization (CDSCO) approval in India-and the evolving competitive landscape. Overall, semaglutide 2.4 mg represents the first and only anti-obesity medication with proven CV benefit in people with obesity without type 2 diabetes (T2D), with important implications for clinical practice and health policy.

## Introduction and background

The global burden of obesity and cardiovascular disease

Obesity has transitioned from a predominantly high-income country problem to a truly global health crisis, with rising prevalence across all regions and age groups. Excess adiposity is now a leading contributor to cardiovascular (CV) disease (CVD), which remains the foremost cause of death worldwide. Recent estimates indicate that 1.95 million CVD deaths were attributable to elevated BMI in 2021, highlighting the scale of the problem [1]. Parallel analyses show that while age-standardized CV mortality has declined over recent decades, the burden specifically attributable to overweight and obesity has increased substantially [2].

In fact, while CV mortality has decreased over the past two decades, overweight- and obesity-related CV deaths have increased threefold [3,4]. The World Obesity Federation further reports that approximately 16% of global ischemic heart disease (IHD) deaths are due to high BMI [5]. These data underscore that obesity is not merely a cosmetic or lifestyle issue but a central driver of global CV morbidity and mortality, demanding targeted therapeutic strategies.

Obesity clusters with a wide range of cardiometabolic comorbidities that collectively magnify CV risk. In metabolic dysfunction-associated steatotic liver disease (MASLD)/metabolic dysfunction-associated steatohepatitis (MASH), up to 75% of affected individuals have obesity [6]. Similarly, obese people are at 1.81 times higher risk of developing chronic kidney disease (CKD) than the non-obese population, and 65%-75% of individuals with primary hypertension have excess body weight [7,8]. Obesity is present in 11%-14% of people with heart failure (HF), in >80% of those with coronary heart disease (CHD), and in 18%-44% of patients with stroke [9-11].

Despite improvements in acute CV care, revascularization, and secondary prevention, the mortality burden attributable to obesity continues to rise. Approximately 70% of deaths related to overweight or obesity are due to CVD [5,12]. This paradox-declining overall CV mortality but rising obesity-related CV deaths-highlights the limitations of current strategies that focus predominantly on downstream risk factors (e.g., blood pressure, lipids, and glucose) without adequately addressing excess adiposity. It also underscores the importance of therapies that can both reduce weight and directly modify CV risk.

Pathophysiological nexus between obesity and CVD

**Figure 1:**
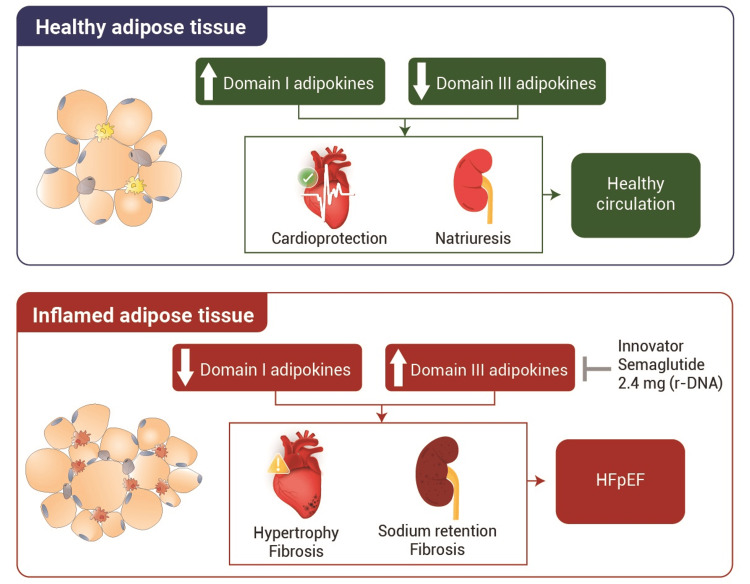
Therapeutic targeting of adipose inflammation: role of semaglutide 2.4 mg (recombinant DNA (r-DNA)). Adapted from Packer [15] (Creative Commons Attribution-NonCommercial-NoDerivatives 4.0 International (CC BY-NC-ND 4.0)) and Ábel and Csobod Csajbókné [16] (Creative Commons Attribution 4.0 International (CC BY 4.0)) and prepared using Adobe Illustrator (Adobe Inc., San Jose, CA, USA). HFpEF: heart failure with preserved ejection fraction

The relationship between obesity and CVD is mediated through a complex network of hemodynamic, metabolic, inflammatory, and neurohormonal pathways. Adipose tissue, particularly visceral fat, functions as an active endocrine organ, secreting adipokines and cytokines that promote insulin resistance, dyslipidemia, endothelial dysfunction, and systemic inflammation (Figure 1) [13]. Epidemiological data suggest that each one-point increase in BMI above normal weight causes a 10% increase in the risk for atherosclerosis and CHD, reflecting a near-linear risk gradient [14].

Obesity confers an increased risk of type 2 diabetes (T2D) and MASLD/MASH, further amplifying CV risk [17]. Mechanistically, obesity drives CVD through multiple converging pathways, including atherosclerosis, arrhythmia, thromboembolism, HF, and valvular disease [13]. This multifaceted pathophysiology provides a strong rationale for interventions that address both weight and broader cardiometabolic dysfunction.

Unmet need in obesity pharmacotherapy

Lifestyle modification remains the cornerstone of obesity management, yet long-term weight loss and CV risk reduction with lifestyle alone are often modest and difficult to sustain. Bariatric surgery offers substantial and durable weight loss with CV benefit but is invasive and accessible to only a minority of eligible patients [18]. Historically, anti-obesity pharmacotherapies have been limited by modest efficacy, safety concerns, or lack of robust CV outcome data [19-21].

Consequently, obesity is a major CV risk factor, and despite current standard of care, residual risk of CV events and excess mortality remain-there is an unmet need for therapies that reduce CV events and support weight management [22,23]. The advent of glucagon-like peptide-1 receptor agonists (GLP-1RAs) with potent weight-lowering and cardioprotective effects has therefore been a major paradigm shift in obesity pharmacotherapy.

Innovator recombinant DNA semaglutide and rationale for review

Semaglutide is a long-acting GLP-1 analogue produced using recombinant DNA (r-DNA) technology in a microbial expression system, followed by peptide purification and site-specific chemical modifications that enhance albumin binding and proteolytic stability [24]. These structural modifications prolong its half-life, enabling once-weekly subcutaneous administration while preserving high affinity for the GLP-1 receptor. As an innovator biologic, semaglutide 2.4 mg (Wegovy®) has undergone extensive characterization of its manufacturing process, quality attributes, and clinical performance across multiple indications [25,26].

Semaglutide has demonstrated a wide range of benefits in the GLP-1 class beyond glycemic control and weight loss for people with cardiometabolic diseases [27]. Building on earlier CV outcome trials (CVOTs) in T2D, the SELECT trial established semaglutide 2.4 mg as the first anti-obesity medication to reduce major adverse CV events (MACE) in people with obesity and established CVD but without diabetes [28,29]. On February 25, 2025, the Central Drugs Standard Control Organization (CDSCO) in India approved Wegovy® (semaglutide 2.4 mg manufactured using r-DNA technology) as the first and only innovator r-DNA semaglutide with an indication to reduce the risk of MACE (CV death, non-fatal myocardial infarction (MI), or non-fatal stroke) in adults with established CVD and either obesity or overweight [30]. This review synthesizes the totality of evidence from randomized controlled trials (RCTs) and real-world studies to interpret the role of semaglutide 2.4 mg in obesity with CVD.

## Review

Methodology

This review was conducted as a narrative evidence synthesis. A literature search was performed in PubMed/MEDLINE, Embase, Google Scholar, and ClinicalTrials.gov from January 2018 to May 2026. Search terms included "semaglutide", "obesity", "cardiovascular disease", "SELECT", "STEP-HFpEF", "major adverse cardiovascular events", "HFpEF", "GLP-1 receptor agonists", and related terms. A supplementary manual search was performed to identify additional relevant evidence from reference lists of included articles, major clinical guidelines, regulatory documents, and recent congress presentations that were considered pertinent to the scope of this review. Also, several findings discussed in this review derive from conference presentations and preliminary analyses that have not yet undergone full peer review and should therefore be interpreted as hypothesis-generating.

Eligible publications included RCTs, observational studies, guideline documents, regulatory publications, and major congress presentations relevant to semaglutide 2.4 mg and CV outcomes in obesity. Studies unrelated to CV outcomes, in vitro and in vivo studies, and non-English publications were excluded. Evidence was synthesized narratively owing to heterogeneity in study designs, populations, and reported outcomes.

Obesity as a driver of CVD

Mechanistic Pathways: How Obesity Drives CVD

Obesity promotes CVD through multiple, overlapping mechanisms. Traditional risk factors such as T2D, hypertension, dyslipidemia, and obstructive sleep apnea are more prevalent and more severe in people with obesity [13]. Atherosclerotic disease manifests as coronary artery disease, peripheral arterial disease (PAD), and cerebrovascular disease, while arrhythmic complications include atrial fibrillation and sudden cardiac death. Thromboembolic events such as deep vein thrombosis and pulmonary embolism are also more frequent [31].

HF represents a major downstream consequence, with both HF with reduced ejection fraction (HFrEF) and HF with preserved ejection fraction (HFpEF) linked to obesity, alongside valvular disease such as aortic stenosis [32,33]. Collectively, these pathways illustrate that obesity is not a single-organ disease but a systemic disorder that accelerates vascular, myocardial, and valvular pathology.

The Obesity-HFpEF Phenotype

HFpEF has emerged as a prototypical obesity-related CV syndrome. Up to 80% of the total HFpEF population lives with overweight or obesity [34]. Increased visceral (intra-abdominal) fat is a strong, independent predictor of decreased physical function in HFpEF [34]. Obesity promotes HFpEF through insulin resistance, systemic inflammation, and hypertension, while impairing cardiac and skeletal muscle function and arterial compliance [35].

The obesity phenotype of HFpEF is characterized by increased epicardial fat, greater biventricular remodeling, increased pulmonary vascular disease, and limited cardiac reserve-associated with higher filling pressures and increased ventricular interaction compared with the non-obese HFpEF phenotype [36,37]. This distinct pathophysiology provides a strong rationale for weight-centric therapies such as semaglutide in this population.

Semaglutide across the cardiometabolic spectrum: mechanism and guideline positioning

Pleiotropic Cardiometabolic Benefits

Semaglutide is a potent GLP-1RA that exerts multiple beneficial effects across the cardiometabolic spectrum. In obesity, once-weekly semaglutide 2.4 mg induces substantial and sustained weight loss, with mean reductions of ~15% in STEP 1 and durable effects over two years in STEP 5 [38,39]. In T2D, semaglutide improves glycemic control with significant reductions in HbA1c and has demonstrated CV benefit in high-risk patients [28,40].

Beyond glycemia and weight, semaglutide has been associated with reductions in MACE in T2D and in obesity without diabetes, early CV benefit, anti-inflammatory effects, kidney protection, improved HFpEF outcomes, favorable effects on MASH, and benefits in PAD [28,29,41-46]. As summarized by Lincoff et al., semaglutide has demonstrated extensive evidence across multiple cardiometabolic conditions in the GLP-1 class beyond glycemic control and weight loss for people with cardiometabolic diseases [29].

Guideline Endorsement Across Indications

Reflecting this broad evidence base, multiple international societies now recommend GLP-1RAs, including semaglutide, across a range of cardiometabolic indications. The 2023 European Society of Cardiology (ESC) guidelines on diabetes, prediabetes, and CVD endorse GLP-1RAs with proven CV benefit in patients with T2D and established CVD [47]. The 2024 ESC guidelines on chronic coronary syndromes (CCS) state that the GLP-1 analogue semaglutide should be considered in CCS patients without diabetes who have overweight (BMI > 27 kg/m²) or obesity to reduce CV mortality, MI, or stroke (Class IIa, Level B) [48].

Other bodies, including the American Diabetes Association (ADA)/European Association for the Study of Diabetes (EASD), European Association for the Study of the Liver (EASL)-EASD-European Association for the Study of Obesity (EASO), American Association of Clinical Endocrinology (AACE), Obesity Canada, Kidney Disease: Improving Global Outcomes (KDIGO), iCARDiO, and American College of Cardiology (ACC)/American Heart Association (AHA), similarly recommend GLP-1RAs and semaglutide in appropriate populations [49-55]. As noted, guidelines recommend the use of GLP-1RAs and semaglutide across a variety of indications [48].

The SELECT trial: design, population, and primary outcomes

Trial Design

SELECT was a landmark CVOT designed to evaluate whether semaglutide 2.4 mg reduces CV events in people with overweight or obesity and established CVD but without diabetes. It was a multicenter, double-blind, randomized, placebo-controlled, event-driven superiority trial enrolling patients aged ≥45 years with BMI ≥ 27 kg/m², prior MI or stroke (≥60 days), or symptomatic PAD, and no history of diabetes (HbA1c < 6.5%) [29,56,57]. Participants were randomized 1:1 to semaglutide 2.4 mg once-weekly subcutaneous or placebo, both on top of standard of care, across 41 countries and 804 sites.

In total, the trial involved 17,604 participants from more than 40 countries, with a median follow-up of ~40 months [29]. The primary endpoint was time to the first occurrence of 3P-MACE (CV death, non-fatal MI, or non-fatal stroke). SELECT thus addressed a critical evidence gap by evaluating CV outcomes in people with obesity and established CVD but without diabetes.

Baseline Characteristics

The SELECT population was representative of high-risk patients with obesity and atherosclerotic CVD (ASCVD). Mean BMI was 33.3 kg/m², indicating Class I-II obesity, and the mean estimated glomerular filtration rate (eGFR) was 82.5 mL/min/1.73 m2. Mean HbA1c was 5.8%, and 66.4% of participants had prediabetes, reflecting a high prevalence of dysglycemia despite the exclusion of overt diabetes. Chronic HF was present in 24.3% of participants, with HFpEF in 12.9%, HFrEF in 7.7%, and unknown HF phenotype in 3.8% [29,56]. These characteristics underscore that SELECT enrolled a population with substantial cardiometabolic burden, in whom both weight reduction and direct CV risk modification are clinically relevant.

Primary Outcome: 20% MACE Reduction

SELECT met its primary endpoint, demonstrating that semaglutide 2.4 mg significantly reduces the risk of MACE versus placebo, both added to standard of care for CV risk factor management, over a mean follow-up period of ~40 months. All three components contribute to this MACE reduction. The hazard ratio (HR) for 3P-MACE was 0.80 (95% confidence interval (CI) 0.72-0.90; p < 0.001 for superiority), corresponding to a 20% relative risk reduction. The benefit was consistent across the components of CV death, non-fatal MI, and non-fatal stroke [29]. Semaglutide’s CV risk reduction varied by race, with the largest effect in Asian individuals (HR 0.64), and smaller yet significant effects in White (HR 0.81) and Black/African (HR 0.87) participants [29]. This result established innovator semaglutide 2.4 mg (r-DNA) as the first and only anti-obesity medication to reduce MACE in people with obesity and established CVD without diabetes, with important implications for guideline recommendations and regulatory approvals.

Reduction in Cardiometabolic Risk Factors

In addition to reducing MACE, semaglutide 2.4 mg produced substantial improvements in cardiometabolic risk factors in SELECT. Participants receiving semaglutide experienced an 11.7% reduction in body weight and a 6.5-cm reduction in waist circumference, reflecting meaningful reductions in overall and central adiposity. HbA1c decreased by 0.31%, despite the absence of diabetes at baseline, and both systolic and diastolic blood pressure were reduced [29].

Inflammatory burden was markedly attenuated, with a 37.8% reduction in high-sensitivity C-reactive protein (hsCRP). Lipid parameters also improved, with increases in high-density lipoprotein (HDL) cholesterol and decreases in total cholesterol, low-density lipoprotein (LDL) cholesterol, and triglycerides [29]. These changes highlight the broad cardiometabolic impact of semaglutide beyond weight loss alone.

Number-Needed-to-Treat Analysis: Demonstrating Broader Clinical Value

A secondary analysis of SELECT evaluated the composite number-needed-to-treat (NNT) for different outcome clusters. At one and four years, NNTs for 3P-MACE were 125 and 58, respectively, corresponding to the 20% relative risk reduction. For an extended composite endpoint (NNT_EXTENDED), NNTs were 49 and 25, while for a cardiometabolic composite (NNT_CKM), NNTs were 20 and 11, reflecting a 41% relative risk reduction [58]. These findings demonstrate that semaglutide provided broader clinically relevant benefits beyond the primary MACE endpoint, capturing the cumulative impact on CV events, cardiometabolic risk factors, and related outcomes [58].

Superiority Among Anti-obesity Medications in CVOTs

When SELECT is viewed in the context of prior CVOTs with anti-obesity medications, semaglutide’s profile is unique. Sibutramine in SCOUT was associated with an increased risk of CV events (HR 1.16), while rimonabant in CRESCENDO (HR 0.97), naltrexone/bupropion in LIGHT/CONVENE, and lorcaserin in CAMELLIA-TIMI (HR 0.97) did not demonstrate significant MACE reduction [19-21,23]. In contrast, semaglutide in SELECT is the only agent to show a statistically significant superiority result for 3P-MACE [29]. This reinforces its status as the first anti-obesity medication with proven CV benefit in obesity without T2D.

CV benefit beyond weight loss: mediation analyses and early benefit

Several findings discussed below derive from conference presentations and preliminary analyses that have not yet undergone full peer review and should therefore be interpreted as hypothesis-generating.

Speed of Benefit: Day 20 and Day 86 Findings

One of the most striking observations from SELECT is the early separation of event curves. A secondary analysis showed that at Day 20, the first statistically significant MACE reduction with semaglutide 2.4 mg versus placebo was observed, and at Day 86, the first sustained statistically significant difference in MACE reduction with semaglutide 2.4 mg was achieved. Importantly, this early MACE reduction preceded meaningful weight loss, suggesting an effect of semaglutide 2.4 mg beyond weight loss [29]. These temporal dynamics imply that mechanisms other than, or in addition to, weight loss-such as anti-inflammatory effects, improved endothelial function, or direct vascular actions-may contribute to early CV risk reduction.

MACE Reduction at Six Months Preceding Meaningful Weight Loss

Further insights come from an analysis presented at the European Congress on Obesity (ECO) 2025. Shiele et al. reported a 41% reduction in MACE within the first six months of randomization (HR 0.59; 95% CI 0.44-0.80), with data confirming that MACE reduction appears even prior to clinically significant weight loss [59]. At this early time point, most participants had not yet achieved ≥5% weight loss, the conventional threshold for clinically meaningful weight reduction. These findings strengthen the hypothesis that semaglutide’s CV benefits are at least partly weight-independent, particularly in the early phase of treatment.

Mediation Analysis: Weight-Independent Mechanisms

A formal mediation analysis presented at ESC 2024 quantified the contribution of weight loss and other measured risk factors to the observed MACE reduction in SELECT. The analysis showed that ~80% of the MACE risk reduction with semaglutide is mediated by factors other than weight loss (body weight mediation: 19.5%; 95% CI −33.0, 110.7), and ~70% of the MACE risk reduction with semaglutide is mediated by factors other than weight loss and measured CV risk factors. The estimated joint mediation of all measured risk factors-body weight, waist circumference, HbA1c, hsCRP, systolic blood pressure, LDL cholesterol, eGFR, and urine albumin-to-creatinine ratio (UACR)-was only 31.4% (−30.1%, 143.6%) [60]. Thus, the MACE risk reduction seen with semaglutide 2.4 mg is not fully explained by the change in body weight or measured CV risk factors, and the exact mechanisms remain to be fully elucidated [60].

Real-world evidence: SCORE and STEER studies

Study Designs and SELECT-Like Population

Real-world evidence (RWE) complements RCT data by evaluating treatment effects in broader, more heterogeneous populations. The SCORE and STEER studies used the Komodo Research Database to identify SELECT-like populations-people with obesity (BMI ≥ 27 kg/m²), age ≥ 45 years, prior MI, stroke or PAD, and no history of diabetes. SCORE was a retrospective, longitudinal, observational cohort study comparing semaglutide users versus non-users, while STEER was a retrospective propensity score-matched cohort study comparing semaglutide versus tirzepatide [61,62]. These designs allow assessment of CV outcomes in routine clinical practice, including comparisons with other incretin-based therapies.

SCORE Results: 57% Reduction Versus Non-users

In SCORE, semaglutide 2.4 mg use was associated with a substantial reduction in CV events. The study demonstrated a 57% reduction in 3P-MACE among semaglutide users compared with non-users in a real-world SELECT-like population [61]. While observational data are subject to residual confounding, the magnitude and direction of effect are consistent with, and even more pronounced than, the RCT findings from SELECT.

STEER Results: 57% Reduction Versus Tirzepatide

STEER provided the first real-world head-to-head comparison of CV outcomes between semaglutide 2.4 mg and tirzepatide in a SELECT-like population. The study showed a 57% reduction in 3P-MACE with semaglutide 2.4 mg compared with tirzepatide in propensity score-matched cohorts [62]. These findings suggest that, in routine practice, semaglutide was associated with lower observed MACE rates than tirzepatide in this observational analysis; however, causal inference is limited by the study design, and prospective CVOT data are not yet available for tirzepatide.

SCORE PRIMARY: Broader Composite MACE Outcomes

In SCORE PRIMARY, a broader high-CV-risk population was evaluated. Semaglutide 2.4 mg use was associated with a 31% lower risk of revised 5P-MACE (MI, stroke, hospitalization for HF, coronary revascularization, and all-cause mortality; HR 0.69; 95% CI 0.62-0.77; p < 0.001) and a 45% lower risk of revised 3P-MACE (MI, stroke, and all-cause mortality; HR 0.55; 95% CI 0.47-0.65; p < 0.001) compared with non-users [26]. These results reinforce the broad CV benefits of semaglutide 2.4 mg across different high-risk populations in real-world settings.

Semaglutide in high-risk CV subgroups

People With Obesity and ASCVD (SELECT Full Population)

In the full SELECT population with established ASCVD, semaglutide 2.4 mg reduced the risk of 3P-MACE by 20% (HR 0.80; 95% CI 0.72-0.90; p < 0.001) over a mean follow-up of ~40 months. As noted, semaglutide 2.4 mg reduced the risk of MACE by 20% across CV death, non-fatal MI, and non-fatal stroke [29]. The benefit was consistent across key subgroups, including age, sex, baseline BMI, and presence of prediabetes, supporting the robustness of the effect.

People With Obesity and Prior Coronary Artery Bypass Grafting (SELECT Subgroup)

A prespecified subgroup analysis presented at the AHA Scientific Sessions 2024 evaluated participants with prior coronary artery bypass grafting (CABG). In this high-risk subgroup, semaglutide 2.4 mg use was associated with 28% lower risk of 3P-MACE in people with obesity with CABG (HR 0.72; 95% CI 0.54-0.95; incidence rate 3.4; absolute risk reduction 2.3%) [63]. These data suggest that semaglutide 2.4 mg provides clinically meaningful CV risk reduction even in patients with advanced coronary disease and prior revascularization.

ESC 2024 Guideline Recommendation for CCS Without T2D

The SELECT findings have been rapidly incorporated into guidelines. The 2024 ESC guidelines for CCS specify that the GLP-1 analogue semaglutide should be considered in CCS patients without diabetes who have overweight (BMI > 27 kg/m²) or obesity to reduce CV mortality, MI, or stroke (Class IIa, Level B). In addition, GLP-1RAs with proven CV benefits are recommended in patients with T2D and CCS to reduce CV events, independently of baseline or target HbA1c and independently of concomitant glucose-lowering medications (Class I, Level A) [48]. These recommendations formally recognize semaglutide as a CV risk-modifying therapy in people with obesity and CCS, irrespective of diabetes status.

CDSCO Regulatory Approval in India (February 2025)

On February 25, 2025, India’s CDSCO granted an additional indication approval for semaglutide 2.4 mg to reduce the risk of MACE (CV death, non-fatal MI, or non-fatal stroke) in adults with established CVD and either obesity or overweight [30]. This approval makes Wegovy® the first and only innovator r-DNA semaglutide 2.4 mg with a CV risk-reduction indication in India, aligning national regulatory practice with global evidence from SELECT and reinforcing the clinical importance of semaglutide in high-risk Indian patients with obesity and CVD.

Wegovy® in obesity-related HFpEF

Pathophysiology: Dysfunctional Adipocytes and HFpEF

**Figure 2:**
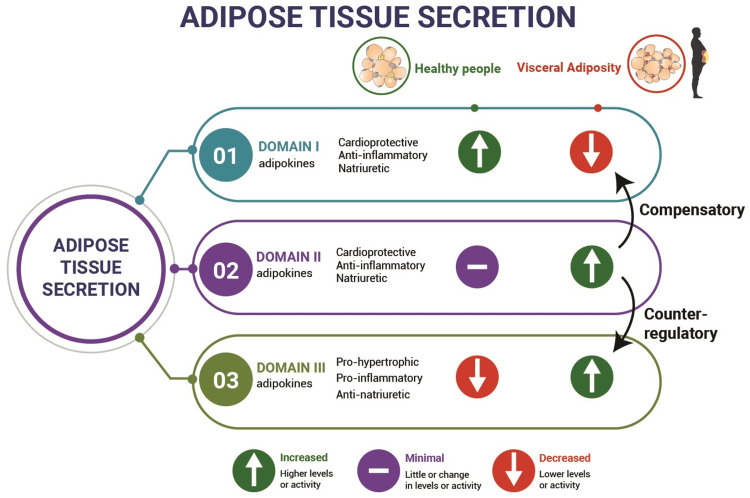
Adipose tissue secretion patterns across adipokine domains in healthy people versus visceral adiposity. This schematic illustrates the differential secretion of adipokines from adipose tissue across three functional domains. Domain I adipokines (cardioprotective, anti-inflammatory, and natriuretic) are increased in healthy individuals but reduced in visceral adiposity, reflecting loss of compensatory signaling. Domain II adipokines (cardioprotective, anti-inflammatory, and natriuretic) remain minimal in healthy states but rise in visceral adiposity as counter-regulatory mediators. Domain III adipokines (pro-hypertrophic, pro-inflammatory, and anti-natriuretic) are suppressed in healthy people but elevated in visceral adiposity, contributing to adverse cardiometabolic effects. Arrows indicate the direction of change (increase, decrease, or minimal change) in each physiological state. Adapted from Packer [15] (Creative Commons Attribution-NonCommercial-NoDerivatives 4.0 International (CC BY-NC-ND 4.0)) and prepared using Adobe Illustrator (Adobe Inc., San Jose, CA, USA).

The pathogenesis of obesity-related HFpEF has been conceptualized in the adipokine hypothesis. In visceral obesity, biological transformation of adipose tissue leads to altered synthesis of adipokines, with Domain III adipokines exerting pro-hypertrophic, pro-inflammatory, and anti-natriuretic effects. These changes drive maladaptive renal effects (anti-natriuretic effect, plasma volume expansion, and renal fibrosis) and maladaptive cardiac effects (cardiac hypertrophy, microvascular dysfunction, and cardiac fibrosis), ultimately leading to increased filling of the left ventricle and decreased ability of the left ventricle to tolerate filling-the hemodynamic substrate of HFpEF (Figure 2) [15].

This framework highlights the central role of dysfunctional adipose tissue in HFpEF pathogenesis and suggests that therapies targeting adiposity and adipokine signaling, such as semaglutide, may have disease-modifying potential. As noted earlier, the obesity phenotype of HFpEF is characterized by increased epicardial fat, greater biventricular remodeling, increased pulmonary vascular disease, and limited cardiac reserve-associated with higher filling pressures and increased ventricular interaction [36]. Increased visceral adiposity promotes insulin resistance, inflammation, and hypertension, while impairing cardiac and skeletal muscle function, arterial function, and physical capacity [64,65]. These observations provide a strong mechanistic rationale for weight-centric therapies in HFpEF and set the stage for the STEP-HFpEF program.

STEP-HFpEF and STEP-HFpEF DM Trial Designs

STEP-HFpEF and STEP-HFpEF DM were parallel, randomized, double-blind, placebo-controlled trials evaluating semaglutide 2.4 mg in people with obesity-related HFpEF, with and without T2D. Both trials investigated the effects of semaglutide 2.4 mg subcutaneously once weekly on physical function, symptoms, and body weight in people with the obesity phenotype of HFpEF with and without T2D. STEP-HFpEF enrolled 529 participants with New York Heart Association (NYHA) Class II-IV, left ventricular ejection fraction (LVEF) ≥ 45%, BMI ≥ 30 kg/m², and no T2D; STEP-HFpEF DM enrolled 616 participants with similar HFpEF criteria but with T2D (HbA1c ≤ 10%). Treatment duration was 52 weeks in both trials [43,44].

Primary Endpoint Results: Dual Endpoint Met

In both trials, semaglutide 2.4 mg met the primary dual endpoint of improvement in HF symptoms/physical limitations and weight loss. Wegovy® met the primary dual endpoint of significant improvements in HF symptoms, physical limitations, and significant weight loss versus placebo. In STEP-HFpEF, the Kansas City Cardiomyopathy Questionnaire clinical summary score (KCCQ-CSS) improved by 19.1 points with semaglutide versus 10.3 points with placebo (estimated difference (ETD) 8.8; p < 0.0001), and body weight decreased by 15.1% versus 2.4% (ETD −12.7%; p < 0.0001). In STEP-HFpEF DM, KCCQ-CSS improved by 16.6 versus 7.9 points (ETD 8.6; p < 0.001), and body weight decreased by 11.0% versus 3.1% (ETD −7.9%; p < 0.001) [43,44]. These results demonstrate that semaglutide 2.4 mg substantially improves symptoms, functional status, and weight in people with obesity-related HFpEF, irrespective of diabetes status.

Secondary Outcomes: Exercise, Inflammation, and Composite Endpoint

Semaglutide also improved exercise capacity and reduced inflammation. Six-minute walk distance (6MWD) increased by 29.0 m versus 8.8 m in STEP-HFpEF (ETD 20.6 m; p = 0.0007) and by 21.5 m versus 3.4 m in STEP-HFpEF DM (ETD 18.1 m; p = 0.008). hsCRP was reduced by ~49.0% and 47.4% with semaglutide versus ~9.1% and 7.9% with placebo (ETR 0.56 and 0.57; p < 0.0001 and p < 0.001) [43,44].

Semaglutide led to 72% and 58% more wins for a hierarchical composite endpoint comprising time to all-cause death, time to and number of HF events, and changes in KCCQ-CSS and 6MWD (stratified win ratio 1.72 and 1.58; both p ≤ 0.001). Thus, semaglutide 2.4 mg significantly improved exercise function and reduced inflammation, compared with placebo [43,44].

Pooled Analysis: Reduction in HF Events and CV Death

A pooled analysis of STEP-HFpEF and STEP-HFpEF DM further clarified the impact of semaglutide on clinical events. Butler et al. reported that semaglutide 2.4 mg improves HF symptoms, reduces physical limitations, and lowers the risk of CV death and HF events in people with obesity with HFpEF. Semaglutide produced approximately twofold greater improvement in KCCQ-CSS versus placebo (~18 versus 9 points; p < 0.0001), a 69% reduction in the composite of CV death or HF events (p = 0.0004), a 73% reduction in HF events (hospitalization or urgent visits; p = 0.0008), and a 25 m improvement in 6MWD (p = 0.0004) [66]. These findings indicate that semaglutide 2.4 mg not only improves symptoms and function but also reduces HF events and CV death in obesity-related HFpEF.

Guideline Recognition

The emerging evidence has been recognized by professional societies. The 2025 ACC Scientific Statement on the management of obesity in adults with HF highlights the emerging evidence of the benefits of semaglutide in individuals with HFpEF and obesity, noting improvements in functional status, symptom burden, and reductions in hospitalization for HF [67]. The Obesity Association 2026 framework designates semaglutide as the only anti-obesity medication with demonstrated benefit (Level A evidence) for reducing HF events in obesity with HFpEF, while tirzepatide is listed as having only potential benefit (Level B) [68]. This further consolidates semaglutide’s unique position in obesity-related HFpEF.

Safety and tolerability

Gastrointestinal Adverse Events Profile

Across SELECT and the STEP-HFpEF trials, the safety profile of semaglutide 2.4 mg was consistent with the GLP-1RA class. Gastrointestinal (GI) adverse events-primarily nausea, vomiting, diarrhea, and constipation-were the most common treatment-emergent events [29,43,44]. These events were generally mild to moderate, dose-dependent, and tended to occur during dose escalation. Gradual titration and patient counseling are therefore important to optimize tolerability.

Discontinuation Rates in High CV Risk and HFpEF Populations

In SELECT, discontinuation due to adverse events was higher with semaglutide than with placebo but remained acceptable in the context of the observed CV benefits [29]. In STEP-HFpEF and STEP-HFpEF DM, discontinuation rates were also higher with semaglutide, largely driven by GI events [43,44]. However, the majority of patients were able to continue therapy, and the net clinical benefit-considering improvements in symptoms, function, weight, and CV outcomes-was strongly favorable.

Special Considerations in HF and Renal Impairment

In HFpEF populations, careful monitoring of volume status, blood pressure, and renal function is warranted, particularly in patients receiving concomitant diuretics or renin-angiotensin-aldosterone system inhibitors. Semaglutide has shown kidney-protective effects in T2D with CKD, and no major safety signals have emerged in patients with mild-to-moderate renal impairment [42,69]. Nonetheless, dose escalation should be individualized, and clinicians should remain vigilant for dehydration or acute kidney injury in the context of GI side effects.

Discussion

Synthesis: Convergence of RCT and RWE

**Figure 3:**
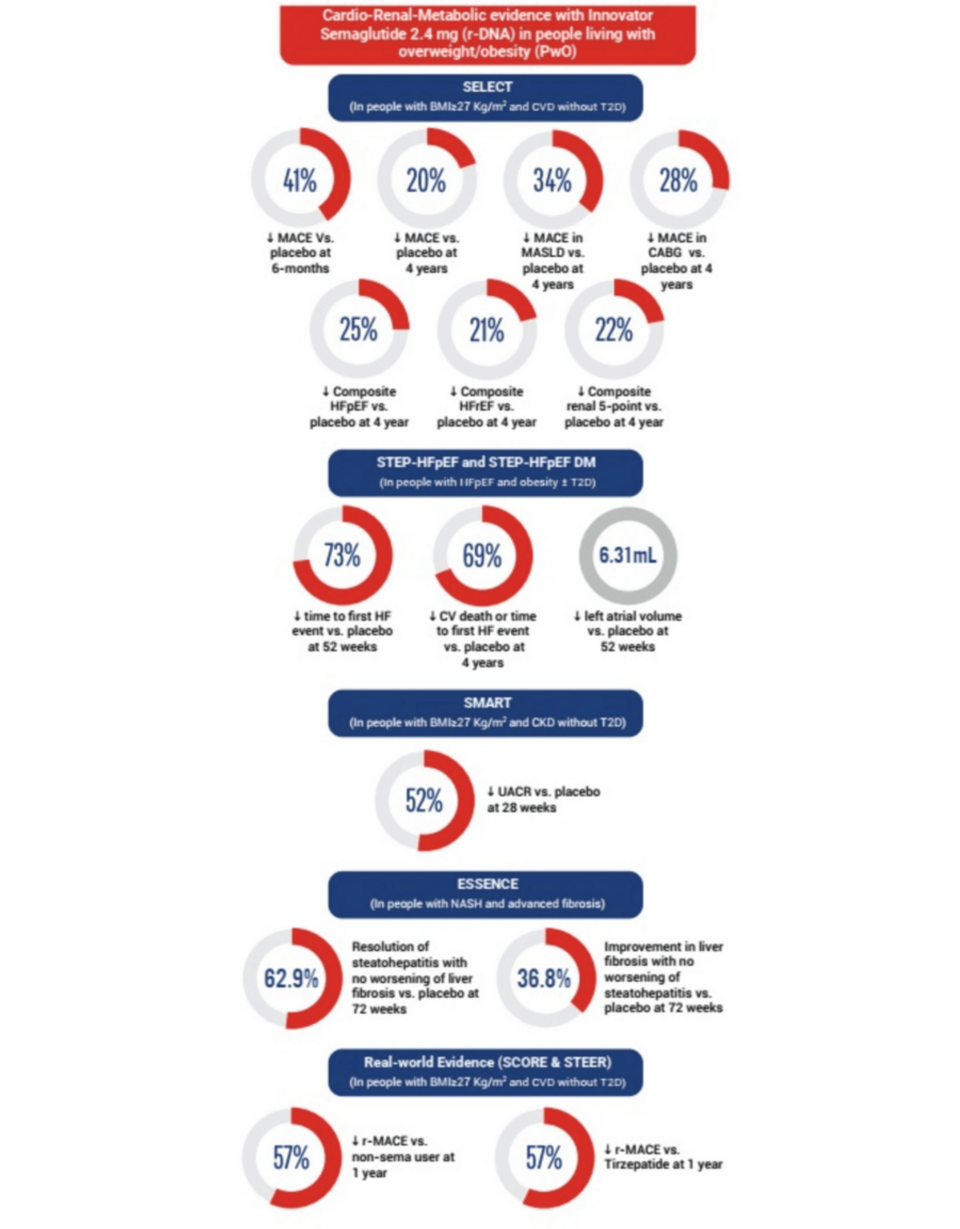
Impact of semaglutide 2.4 mg on MACE, HFpEF, CKD, and MASLD: insights from randomized trials and real-world studies. References [26,28,29,41-45,61,62,66,69,70] MACE: major adverse cardiovascular events; HFpEF: heart failure with preserved ejection fraction; CKD: chronic kidney disease; MASLD: metabolic dysfunction-associated steatotic liver disease; r-DNA: recombinant DNA; CVD: cardiovascular disease; T2D: type 2 diabetes; CABG: coronary artery bypass grafting; HFrEF: heart failure with reduced ejection fraction; HF: heart failure; CV: cardiovascular; UACR: urine albumin-to-creatinine ratio Figure prepared using Adobe Illustrator (Adobe Inc., San Jose, CA, USA).

The totality of evidence (Figure 3) from SELECT, STEP-HFpEF, STEP-HFpEF DM, and real-world studies such as SCORE and STEER paints a coherent picture: semaglutide 2.4 mg delivers robust CV risk reduction, substantial weight loss, and meaningful improvements in symptoms and function across a spectrum of high-risk cardiometabolic populations. SELECT established a 20% reduction in 3P-MACE in people with obesity and established CVD without diabetes, while STEP-HFpEF trials demonstrated improvements in KCCQ-CSS, 6MWD, HF events, and CV death in obesity-related HFpEF [29,43,44,66]. Real-world data from SCORE and STEER corroborate and extend these findings, showing large relative risk reductions in MACE compared with non-users and tirzepatide [26,62].

Weight-Independent CV Benefit Hypothesis and Clinical Implications

Mediation analyses and early event-curve separation suggest that semaglutide’s CV benefits are not solely attributable to weight loss. As Colhoun et al. showed, only ~20% of the MACE reduction is mediated by body weight and ~30% by all measured risk factors combined, implying that the MACE risk reduction seen with semaglutide 2.4 mg is not fully explained by the change in body weight or measured CV risk factors [60]. Early benefits observed by Day 20 and within six months, before clinically meaningful weight loss, further support this hypothesis [29,59].

Clinically, these findings suggest that semaglutide 2.4 mg may offer benefits beyond weight reduction and could contribute to CV risk reduction in people with obesity and established CVD, including those without diabetes. As evidence continues to evolve, these data may inform future treatment strategies, reimbursement decisions, and guideline recommendations.

Gaps: Ongoing Trials With Other Agents

While semaglutide currently holds a unique position as the exclusive anti-obesity medication with proven CV benefit in obesity without T2D, the landscape is evolving. Ongoing CVOTs such as SURMOUNT-MMO (tirzepatide), REDEFINE-3 (CagriSema), and SYNCHRONIZE-CVOT (survodutide) will provide critical comparative data [71-73]. Until these results are available, semaglutide remains the benchmark for CV risk reduction in obesity pharmacotherapy (Table 1).

**Table 1:** Comparative summary of major cardiovascular outcome trials (CVOTs) evaluating anti-obesity medications. 3P-MACE: cardiovascular death, non-fatal myocardial infarction, and non-fatal stroke; 5P-MACE: all-cause death, non-fatal myocardial infarction, non-fatal stroke, coronary revascularization, or heart failure events; MACE+: myocardial infarction, stroke, cardiovascular death, hospitalization for unstable angina, heart failure, or coronary revascularization; CV: cardiovascular; HF: heart failure; HR: hazard ratio; CI: confidence interval; MI: myocardial infarction; CVOT: cardiovascular outcome trial; T2D: type 2 diabetes

Trial	Intervention	Primary outcome	Trial status	HR (95% CI) for primary outcome
SCOUT [20]	Sibutramine	3P-MACE	Completed	1.16 (1.03-1.31); p = 0.02
CRESCENDO [21]	Rimonabant	3P-MACE	Terminated prematurely (safety concerns)	0.97 (0.84-1.12); p = 0.68
LIGHT [23]	Naltrexone/bupropion	3P-MACE	Terminated prematurely (study integrity compromised)	Interim: 0.88 (0.57-1.34)
CAMELLIA‑TIMI [19]	Lorcaserin	3P-MACE	Completed	MACE+: 0.97 (0.87-1.07); p = 0.55
SELECT [29]	Once-weekly semaglutide 2.4 mg	3P-MACE	Completed	0.80 (0.72-0.90); p < 0.001
SURMOUNT-MMO [72]	Tirzepatide	5P-MACE	Ongoing	N/A
REDEFINE-3 [71]	CagriSema	3P-MACE	Ongoing	N/A
SYNCHRONIZE-CVOT [73]	Survodutide	5P-MACE	Ongoing	N/A

Implications for Clinical Practice and Health Policy

The convergence of robust RCT data, supportive RWE, and guideline endorsements argues for a re-framing of obesity management in cardiology and internal medicine. For patients with obesity and established CVD, particularly those with CCS or HFpEF, semaglutide 2.4 mg should be considered as part of comprehensive CV risk reduction, alongside statins, antiplatelets, renin-angiotensin system blockers, SGLT2 inhibitors, and lifestyle interventions. From a policy perspective, the demonstrated reductions in MACE, HF events, and mortality suggest that broader access to semaglutide could yield substantial public health and economic benefits, especially in countries with high burdens of obesity and CVD.

Limitations

This review has several limitations that should be considered when interpreting the findings. The pivotal SELECT trial enrolled individuals with overweight or obesity, established CVD, and no diabetes, which may limit the generalizability of its findings to broader patient populations. In addition, participants in several of the key clinical trials were predominantly White, potentially limiting applicability across diverse ethnic and geographic groups. Although the available safety data are reassuring, long-term safety and effectiveness beyond the currently reported follow-up periods remain under evaluation. Furthermore, real-world studies such as SCORE and STEER were observational in nature and are therefore subject to residual confounding, selection bias, and other inherent limitations despite statistical adjustment. Several findings discussed in this review are derived from conference presentations and preliminary analyses that have not yet undergone full peer review and should therefore be interpreted with caution. Practical considerations, including treatment access, reimbursement policies, and cost, may also influence the implementation of semaglutide in routine clinical practice. Finally, as this was a narrative review, a formal risk-of-bias assessment and quantitative evidence synthesis were not performed, which may limit reproducibility and introduce the potential for selection bias in the interpretation of the available literature.

## Conclusions

Obesity is a major contributor to morbidity and mortality and remains an important therapeutic target in the prevention and management of CVD. Emerging evidence supports the role of semaglutide 2.4 mg as a comprehensive cardiometabolic intervention that addresses excess adiposity while influencing the broader spectrum of obesity-related cardiometabolic risk. Its relevance across obesity-associated CV conditions, including established CVD and HFpEF, reflects the evolving paradigm of obesity management from a weight-centric approach to a more comprehensive CV risk-reduction strategy.

The convergence of clinical evidence, real-world observations, guideline updates, and regulatory approvals reflects a growing recognition of semaglutide as an important component of contemporary cardiometabolic care. As the understanding of obesity and its CV consequences continues to evolve, semaglutide is poised to play an increasingly important role in comprehensive cardiometabolic management, with the potential to improve clinical outcomes and reduce the burden of obesity-related complications.
